# P02.65. Chiropractic, acupuncture and oriental medicine, and massagec services in two geriatric facilities: outcomes of 1033 patient visits

**DOI:** 10.1186/1472-6882-12-S1-P121

**Published:** 2012-06-12

**Authors:** K Westrom, R Evans, C Vihstadt

**Affiliations:** 1 Northwestern Health Sciences University, Bloomington, USA

## Purpose

There is insufficient information published on the topic of complementary and alternative medicine (CAM) clinical services within geriatric settings. The purpose of this presentation is to describe results of a CAM demonstration project carried out within two geriatric facilities. Acupuncture and Oriental medicine (AOM), chiropractic, and massage clinical services were integrated into the facilities and data systematically collected. We prospectively gathered information on all patient visits provided by the CAM clinicians.

## Methods

The project occurred at an assisted living (AL) complex, and a long term care (LTC) facility which consisted of skilled nursing beds and a transitional care unit (TCU). Chiropractic, AOM, and massage clinical services were each provided 16 hours weekly. Treatment notes designed for data collection were utilized. A project manager oversaw transfer of nonidentifiable patient information and data integrity. Outcomes collected included patient demographics, self-rated pain, main complaint, quality of life (QOL), components of the treatment encounters, and side effects and adverse events.

## Results

Eighty-two patients received CAM clinical care. A total of 1033 treatment visits were provided (366 AOM, 338 chiropractic, and 329 massage). The number of visits per patient ranged from one to 92. There were no serious adverse events. Outcomes of pain, QOL, treatment components, and side effects will be discussed.

## Conclusion

CAM clinical services were safely provided to predominantly geriatric patients in settings not typical for CAM clinicians. Further discussion on data collection in geriatric settings will be part of this presentation.

